# Professional Etiquette

**Published:** 1864-06

**Authors:** A. Lawrence


THE
DENTAL REGISTER OF THE WEST.

VOL. XVIII.] JUNE, 1864. [No. 6.

### Original Essays and Communications.

### PROFESSIONAL ETIQUETTE.

Read before the Lowell Dental Association, May 5th.

BY A. LAWRENCE.

The subject of professional etiquette is an important and delicate one to the dentist.

The dentist is no longer an isolated worker. His pursuit is no longer a mere mechanical affair, incident to half a dozen different handicrafts, as it was formerly, such as the jeweler, silversmith, barber, and even blacksmith, but a distinct, well-organized and numerously followed profession, with its journals, savans and colleges.

Although among the youngest professions, it is admitted to be second to none in importance to the world of *civilized* man, for however much civilization and social progress may improve *other* things, they appear to damage the teeth.

The simple habits and inartificial diet of the savage cause him to retain excellent molars and incisors, while in civilized society the teeth are among the first organs of the body to exhibit the deleterious effects of artificial and unnatural modes of living.

While Americans are reputed to have the worst teeth of all the civilized nations, they are also reputed to be the fastest eaters, and to spend the shortest time at the table.

One good result has grown out of the wide prevalence of defective teeth amongst us. It has enabled the American dentist, by universal consent, to take the first rank in his profession, distancing his old world rivals of England, France and Germany, so that abroad dentistry is regarded as an American art. The human face divine is almost as blank and void of expression without teeth as without eyes. Without teeth, the smile, which is an exclusively *human* characteristic, is destitute of all sweetness and cheerfulness, and becomes a vacant, unmeaning yawn.

Without the proper complement of sound and healthy teeth, mastication and pronunciation can be but poorly and unsatisfactorily performed. A toothless Cicero, Choate, or Everett, if there were no dentist to come to his assistance, would find his vocation gone.

That most agonizing class of diseases which affect the nervous system, are intimately connected with the teeth, and lie within the domain of the dental practitioner.

Odontology is in fact a science of the first importance to the well-being and comfort of every highly refined community. It has been well said that the time is gone by when medical men looked upon dentists as mere mechanical operators, and therefore denied them their confidence and respect. Every general practitioner, it has been remarked, if consulted by patients suffering under certain classes of disease, as indigestion, neuralgic affections, &c., will recognize the important aid to be derived from the skillful services of an intelligent and conscientious dentist. "The insalivation of the food is strictly a mechanical operation," says an eminent Western dentist. "Its trituration and commingling with the secretions, its preparation into a *bolus*, and its deglutition, are all purely of a mechanical nature; and a loss of those implements used in any of these processes of course renders such process defective or impossible. As well might the miller expect a double extra quality of flour, his nether mill-stone being missing, as to expect a mouth, deprived of *grinders*, to prepare proper digestible food for the stomach."

Now the province of the dental art is to supply a substitute for the loss of the natural teeth, and just so far as this can be done with success, should it be estimated and receive the meed of favor which is due. No longer ago than 1820, according to Appleton's *Encyclopedia* there were only one hundred dentists in the United States, all told; now they are numbered by thousands.

In fact the surgeon-dentist now finds himself not an isolated being, but a member of a numerous and most respectable fraternity, with duties and relations toward his professional brethren and the public which he can not ignore. A member of the dental profession might formerly wear his mantle of secresy and keep what knowledge he possessed securely locked in his own bosom, but he can do so no longer. Isolation *now* means weakness, ignorance and inefficiency. Association and associated effort is the law of this democratic age of the world. By association and the interchange of ideas and discoveries, each member of a profession, however humble, becomes enlightened by the concentrated wisdom of all the rest.

I have said that Professional Etiquette is an important and delicate subject to the dentist, for it defines the line of conduct which he should pursue toward his professional brethren and medical men, and in his intercourse with his patients and the public. Professional envy and jealousy are as old as the world. Hesiod, one of the most ancient poets, speaks of singer envying singer, and potter being jealous of potter.

This is human nature in all times and everywhere. A fair, generous rivalry is not to be complained of. It is, in fact, commendable—a public benefit. But selfishness and the love of filthy lucre, which were never more rife than now, in this purely commercial age, are too apt to make men utterly regardless of means, so they can attain, in the most expeditious manner, to the universally desired end; to-wit: a well-filled pocket.

A distinguished dentist, writing upon the subject of professional ethics, says: "We are happy to notice and record the fact, that the most intelligent, honorable and high-minded gentlemen of our profession are willing to acknowledge merit whenever and wherever met with. Indeed, to accord the meed of praise, when the evidence of faithfulness and superior operative quality is immediately under one's inspection, will go much farther to establish, in the minds of those seeking our counsel, confidence in us, and command from them a recognition of a high order of dental ability and acquirement, than the opposite course, and such acts must ever yield a rich harvest of satisfaction and conscientious delight for having done to our professional brother that which we, under like circumstances, would wish to receive at his hands, especially if deserving, and if not, to let a discretionary silence mark the interview.

"It is to be most devoutly hoped, that the practice of general censure is fast passing away. The practice of an indiscriminate condemnation of all operations found in the mouths of persons, whose great misfortune seems to have been in falling into the hands of *other* dentists, is, to speak in the mildest terms, highly censurable, and the person habitually guilty of it should be shunned as the plague or the pestilence."

We sometimes hear, says a distinguished authority, the following: "I dread to change dentists, for I expect to have every filling removed that has been done in my mouth." Something similar is by no means uncommon, and evidently the practice alluded to is indulged in to some extent, or such expressions would never find their way into the aphorisms of any community.

The same writer says: "Professional consultation or advice is as frequent as professional interviews, and very often an operator is called upon, or, if not solicited, the prerogative is oftentimes assumed, to pass judgment upon the work of a professional brother. Many times this is an imperative duty, and it should be performed without exhibiting any malignant *animus* compromising the operator, in remarks calling in question the quality of the operation at the time of its performance. This is not called for, neither is the act justifiable, and no honorable gentleman will be guilty of such a dereliction of good breeding. A plain statement of facts touching the matter under consideration should be made, and this at the time the advice is asked. But it may be asked, should never a word be said in the presence of a patient derogatory to an operator? Most assuredly not. We have to do with the *present* state of the teeth and their artificial appliances, and not with the dentist. The rule should be always to be courteous toward brethren (quacks and humbugs, of course, excepted)."

In a debate upon the subject of professional etiquette, at a national convention of dentists, it was said, "In our judgments of other practitioners, let us be guided by the highest sense of justice and honor. In many cases silence is the better part of valor, as there are oftentimes circumstances over which the operator can have no control, which may affect the quality of the work. Professional etiquette often requires us to descend from our lofty eminence, and consult with those we may consider our inferiors; but who of us has not imperfections? and even a bungler should receive consideration if he is trying to elevate himself. Then it is he is entitled to the respect of every member of the profession. True etiquette goes even farther and requires silence upon other faults; even when justice would seem to demand exposure. It prevents those significant winks and nods which often convey so much; and all public criticism upon a brother's spelling or writing. Selfishness and envy must be thrown aside before we can hope to attain perfection."

Under this head of professional etiquette the personal habits of the dental operator are important and pertinent subjects of consideration. While he is bending over his fair patient, endeavoring to purify and renovate her mouth, his *own* should not be redolent of tobacco and whiskey.

I do not propose to go into a discussion of the general utility or inutility of tobacco. I think it is at least proper to urge, in a discourse of this kind, dentists not to use it, except at such times as to allow the evidences of it to dissipate themselves before coming in contact with their patients. The fumes of tobacco are unpleasant to most ladies, a class with whom we have more to do than any other. That alone should be a sufficient reason for not using it. The engagements with us, of our patients in general, are of a sufficiently unpleasant nature, and we should strive, therefore, to render all our surroundings as unobjectionable as possible, for two reasons. The first, and by far the most worthy of consideration, is, *their* comfort; the second is for our own pecuniary advantage. In his premises, his instruments and in his own person, the dentist should see that the utmost cleanliness prevails. The advantages of personal appearance, of an intelligent physiognomy and gentlemanly bearing, are too palpable in the prosecution of any profession to require more than a passing notice.

The really able, well-instructed and conscientious dentist will scorn any other than legitimate means to raise himself in his profession. He will let his works herald his praise, and never resort to any unprofessional means of getting up a factitious, pseudo reputation. He will be above it. The ignorant and unreflecting may be caught by audacious pretenses and flaming advertisements, but the judicious, *never*.

The purely advertising dentist, like "the *advertising* physician," is generally a quack or charlatan. Of course it is proper enough to reach the public by publishing one's professional card. That does not necessarily make one obnoxious to the stigma of being an advertising dentist.

The universal ability to read, which characterizes Amercans, and the universal diffusion of newspapers, have enabled a set of pill-mongers and bogus medical men, remarkable for nothing but their impudence and lack of all moral principle, to acquire immense fortunes among us by a system of persistent and lying advertisement. That such means should be effective for a while, until the absurdity and hollowness of the pretenses set up should be ascertained by actual trial, is not remarkable, but that such lying appeals should *continue* to gull the public *is* remarkable.

It would seem that *the ability to read*, without which the contemptible advertising teeth-tinkers and pill-mongers could not snare the public, ought to bring with it too much good sense to render its subjects the dupes of such shallow and impudent pretenders and liars; but it does not. A writer in the *Dental Times* says: "There are always a set of impostors and would-be dentists who have recourse to printers' ink (the devil of course has something to do with it), to bring themselves into notice; for if they depended upon merit, on which the true professional man solely relies, their hopes would never be realized; but unfortunately for the people, their flaming advertisements inveigle persons who are not educated to a proper appreciation of dental operations until, alas, too late. Now if their *pockets* were alone the sufferers, there would not be the same cause of complaint, but unfortunately for the poor victim, he or she may have received a permanent injury, perhaps a constitutional one, which may not at once be developed, but nevertheless will sooner or later appear when possibly past all remedy."

If the public could only understand the difference between a good and a bad operation in dentistry, they certainly would pay more attention to their teeth; they would at once see the value and importance of consulting the best operator, whose merits have gained him a reputation and on whom they can rely, and rest assured that what operations are performed, are done in the most thorough and practical manner—and not the miserable quack whose pockets are freely bled by the job printer and sign painter, to make himself known, whose operations are not only a disgrace to himself (if that were possible), but to the science of dentistry.

The foregoing strictures may seem severe, but they are fully warranted. Of course I am not objecting to a proper style of advertisement. It is the usual and most effective method of bringing one's business under the notice of the public, which is resorted to by almost every one in this country of newspapers. But I do object to the quack advertisement got up as a lure and cheat, full of sound and falsehood and pretension, and reflections either direct or by inference upon respectable rivals. I object to that style of advertisement which claims to afford better services for a less consideration than any one else can or ought to do. No one should know better than the dentist himself what his services are worth, and when he proposes to render such service for half what any respectable operator would charge, the inference is that his demands are in strict conformity to the value of his skill. If he estimates his services to be of little worth, the public should do the same. It becomes a question of veracity, but *not* of professional depravity.

I also object to the practice of dentists making exhibitions of gew-gaw fancy work, whether made by themselves, by others for them, or borrowed for the occasion, at fairs and cattle shows, and the fact of any notice being taken of such trash being made the burden of all future professional advertisements, notices and cards. It is simply *unprofessional*, and savors strongly of the quack—the inference being that such parties wish the public to understand that the exhibitor can do something which no one else can do. Show work is no evidence of professional ability.

It militates adversely to the welfare of dental science to admit into our offices as students men, young or old, who are not possessed of even the rudiments of a common school education, and attempt to qualify them to practice the profession in less time than would be required to learn to make a horse shoe. Yet such things are by no means uncommon, and three months is regarded as a long pupilage by many who claim respectability as dental operators. Now I respectfully submit that at this time, with the facilities we have for acquiring a knowledge of the profession, no dentist is excusable for the fraud he perpetrates upon the public by permitting an ignorant blockhead to enter the ranks of dentistry.

Nor is the poor creature thus graduated exempt from the fraud. His preceptor has damaged *him* as well as the public, in not pointing out to him the true course to be pursued—in not advising him to first obtain a general education, as precedent to a thorough professional course of studies. The difficulty or damage is not confined even to the parties enumerated. The whole profession is involved more or less in the reputation of such half-made dentists, and true etiquette denies the propriety of such conduct on the part of any member of a profession, as shall injure the standing of all or any of the rest. Dentists are no more exempt from faults than other men, nor should we expect them to be. Among the failings of human nature there is none more glaring—more fraught with all that is mischievous and wicked—than that of detraction. Does a patient ask an opinion respecting an operation performed by another operator, the answer is too apt to be given in such words or manner as to convey the impression that there has been some cheating or bungling done. A rival's reputation, either professional or moral, is not sacred in the hands of such calumniators. "Oh, Dr. —— is a splendid operator, but he don't finish his work so much as some do. Well, I don't like to say any thing against any man, but others say he is a little rough. You must not ask me any thing about him; ask the ladies." Another very religiously persists in defending the character and professional merits of his compeer, the more so as they have never been called in question. The manner, tone and emphasis, however, convey his meaning. "Oh, no, he don't drink; he don't abuse his wife; he don't play for money; he is a good operator, &c., &c. Oh, well, if you want this work done, I'll do it first rate, beside Dr. —— has moved away." All these and many other verbal means are resorted to every day and almost every where to injure or cast obloquy upon the reputation of a professional brother. Now, I ask, do such things benefit any body? Does the man guilty of detraction, either by word or deed, merit the confidence of the community, and does he fully understand and appreciate all that is applied in the simple word etiquette? There are many violations of professional ethics, which time and your patience forbid that I should even allude to; I therefore refrain.

As surgeon dentistry may now fairly claim to rank among the learned professions, any thing which smacks of the empiric should be avoided by the dental practitioner. A member of a learned profession should be entirely correct in his deportment and in his thoughts. No lurking meanness should poison his impressions of justice, nor compromise his conduct. He should aim to improve himself in every thing good or profitable, and to avoid every thing bad or positively evil. Individual reformation leads to the improvement of the whole, and thus, I respectfully submit, shall we most surely attain the desired end and aim of this Association.

Gentlemen, having extended these remarks far beyond my original design, and farther, perhaps, than has been profitable and interesting to you or creditable to msyelf, I take leave of the subject with the sincere hope that if you can not approve either the effort or the sentiments advanced, you will at least not censure the endeavor to discharge a seeming duty.

